# Impact of the COVID‐19 Pandemic on Child Development and Caregiving: A 7‐Year Repeated Cross‐Sectional Study of 3‐Year‐Old Children in Kobe City, Japan

**DOI:** 10.1002/brb3.71434

**Published:** 2026-04-22

**Authors:** Kazuki Suemune, Hiroshi Yamaguchi, Masahiro Nishiyama, Yuki Kyono, Aoi Kawamura, Shizuka Oikawa, Takumi Imai, Sae Murakami, Hiroki Mishina, Kandai Nozu, Hiroaki Nagase

**Affiliations:** ^1^ Department of Pediatrics Kobe University Graduate School of Medicine Kobe Japan; ^2^ Department of Neurology Hyogo Prefectural Kobe Children's Hospital Kobe Japan; ^3^ Clinical and Translational Research Center Kobe University Hospital Kobe Japan; ^4^ Kobe City Child and Family Bureau Kobe Japan

**Keywords:** COVID‐19, motor skills, social interaction

## Abstract

**Introduction:**

Social restrictions during the coronavirus (COVID‐19) pandemic have resulted in children spending more time with their families and having fewer opportunities to attend nursery schools or therapeutic facilities. Few studies have examined the impact of the COVID‐19 pandemic on the development and parenting environments of 3‐year‐old children. In this study, we aimed to examine the impact of the COVID‐19 pandemic on fine and gross motor skills and language development, including comprehension and communication, in 3‐year‐old children. The secondary objective was to investigate their impact on caregiving environments.

**Methods:**

This repeated cross‐sectional study was conducted at age 3 years using data from a longitudinal birth cohort. We analyzed data from routine 3‐year‐old health examinations conducted in Kobe City, Japan, between April 2014 and March 2021. Multivariable logistic regression models were used to assess the association between COVID‐19 birth cohorts and developmental and caregiving outcomes among 3‐year‐old children. In total, 62,192 children (3 years, 51.4% males; majority Japanese) were categorized into three birth cohorts: pre‐ (from 4/1/2014 to 3/31/2017; *n* = 29,421), partial‐ (from 4/1/2017 to 3/31/2020; *n* = 25,202), and post‐COVID‐19 (from 4/1/2020 to 3/31/2021; *n* = 7569).

**Results:**

The prevalence of impaired gross motor skills and language comprehension was significantly higher in the post‐COVID‐19 group than in the pre‐COVID‐19 group (gross motor skill: 0.7% vs. 0.5%, OR 1.41, 95% CI 1.02–1.95, *p* = 0.04; language comprehension abnormalities: 3.6% vs. 2.1%, OR 1.72, 95% CI 1.49–1.99, *p* < 0.001). However, medical evaluations conducted by physicians showed no differences in fine motor skills. Caregiver questionnaires showed impaired verbal and communication skills, as well as reduced interactions with peers and relatives, in the post‐COVID‐19 group (limited vocabulary growth: 1.0% vs. 0.7%, OR 1.32, 95% CI 1.01–1.72, *p* = 0.04; inability to formulate three‐word sentences: 4.4% vs. 3.4%, OR 1.26, 95% CI 1.11–1.43, *p* < 0.001; inability to state the names of their playmates: 5.5% vs. 3.9%, OR 1.40, 95% CI 1.25–1.57, *p* = 0.003). However, no definitive conclusions regarding motor skills could be drawn from our findings. Although caregiving assistance decreased significantly in the COVID‐19 group, comparable trends were observed in the pre‐COVID‐19 group.

**Conclusion:**

These findings indicate that the COVID‐19 pandemic was associated with changes in language development and social interactions, possibly reflecting decreased opportunities for social engagement outside the family.

## Graphical abstract

This retrospective, repeated cross‐sectional at 3 years derived from a longitudinal birth cohort utilized data from 3‐year‐old health examinations conducted in Kobe City, Japan. The COVID‐19 pandemic was associated with changes in language development and social interactions, possibly reflecting decreased opportunities for social engagement outside the family.

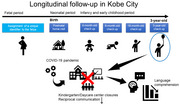

## 1 Introduction

Neurodevelopmental processes that occur during infancy and early childhood are shaped by genetic, environmental, and experiential factors (Kundakovic and Champagne 2015). Collectively, these factors contribute to the acquisition of developmental milestones. Developmental milestones in infants and young children are categorized into six domains: gross motor, fine motor, language, cognitive, social, and emotional development (Scharf et al. 2016). The first 3 years of life are marked by remarkable advances in these domains. In Japan, health checkups for 3‐year‐old children are mandated by law and designed to assess the physical and psychological development, detect diseases early, provide parental guidance on child‐rearing, and connect families with healthcare services.

The coronavirus disease 2019 (COVID‐19) pandemic, which began in late 2019, has profoundly impacted many aspects of daily lives worldwide (Phelan et al. 2020; Schady et al. 2023). Children in the earliest stages of development, particularly those born during or shortly before the pandemic, experienced substantial disruptions in access to fundamental services, including routine childhood immunizations and early educational opportunities, as well as in interactions with others outside their immediate household (Schady et al. 2023; Furuse et al. 2020; Yamaguchi et al. 2022).

Following the first reported case of COVID‐19 in Japan on January 16, 2020 (Furuse et al. 2020), elementary, junior high, and high schools were closed on March 3, 2020 (Yamaguchi et al. 2022). A state of emergency was declared on April 9, 2020, and remained in effect until May 25, during which time many people, including children, refused to go outdoors. Schools resumed activities in June 2020; however, practices such as avoiding social interactions and maintaining good hygiene, including wearing masks, continued to be recommended. On March 13, 2023, the Ministry of Health, Labor, and Welfare in Japan established that wearing masks should be based on individual judgment and respect for personal choices. However, owing to the prolonged period of mask use and social distancing, some individuals continued these practices for years, leading to changes in household and school lifestyles (Hangai et al. 2022). Social restrictions during the COVID‐19 pandemic have resulted in children spending more time with their families and having fewer opportunities to attend nursery schools or therapeutic facilities. Studies have revealed the impact of the COVID‐19 pandemic on the mental health, neurodevelopment, physical health, and maternal healthcare situation of children (de Figueiredo et al. 2021; Huang et al. 2021; Kishida et al. 2021; Ferrari et al. 2022; Hessami et al. 2022; Sato et al. 2023; Aggarwal et al. 2024; Matsuo et al. 2024; Okubo et al. 2024). Children born after the COVID‐19 pandemic may have had reduced interactions with peers. Furthermore, since caregivers and siblings wore masks, they may have struggled with language learning or reading facial expressions. In addition, social distancing recommendations also encouraged maintaining distance even among young children. These factors are expected to potentially affect their motor and language development as well as their social and emotional skills.

Several studies that included 3‐year‐old children have reported the following effects: decreased developmental milestone (Romem et al. 2024), reduced fine motor skills (Choi et al. 2024), declined language and cognitive development (Choi et al. 2024; Perrigo et al. 2025), delay in communication (Choi et al. 2024; Lee et al. 2024; Johnson et al. 2024), reduced social interaction (Choi et al. 2024; Lee et al. 2024), reduced selfcare skills (Choi et al. 2024), decreased problem‐solving (Johnson et al. 2024), and worsening of sleep quality (Di Giorgio et al. 2021). Similarly, a meta‐analysis that included 3‐year‐old children also reported a significant association with the risk of communication delay among children during the COVID‐19 pandemic (Hessami et al. 2022; Penna et al. 2023), as well as a decrease in child‐emotional behavioral functioning (Specht et al. 2021) and maternal mental health (Penna et al. 2023).

To the best of our knowledge, no large‐scale cohort studies have used data from specific 3‐year‐old health checkups to examine the impact of COVID‐19 on developmental outcomes in this age group; consequently, the effects remain unclear. We aimed to examine the impact of the COVID‐19 pandemic on the growth and development of children born before and after the pandemic by comparing data from 3‐year‐old health checkups in Kobe City. The secondary objective was to investigate changes in caregivers' mental well‐being and support environments associated with the pandemic.

## 2 Methods

### 2.1 Study Design

This was a repeated cross‐sectional analysis conducted using data obtained when participants were aged 3 years from a longitudinal birth cohort. We analyzed data from routine 3‐year‐old health examinations conducted in Kobe City, Japan, between April 2014 and March 2021. The study was approved by the Kobe City Ethics Committee (approval number: 4–8). All procedures were performed in accordance with the relevant institutional guidelines and regulations, as well as the Code of Ethics of the World Medical Association (Principles of the Declaration of Helsinki). Since this study was a retrospective analysis based solely on anonymized data, the requirement for obtaining individual informed consent was waived. Instead, in accordance with the Ethical Guidelines for Medical and Biological Research Involving Human Subjects, an opt‐out notice was posted on the Kobe City website (https://www.city.kobe.lg.jp/a86732/kosodate/maternity/kenshin/kenkyu.html) and Kobe University website (https://www.med.kobe‐u.ac.jp/pediat/research/pediatrics1.html#top3). All procedures in this study were performed in compliance with the approved ethical guidelines.

#### 2.1.1 Health Examination of 3‐Year‐Olds in Kobe City

The 3‐year‐old health examinations in Kobe City consist of the following components: medical questionnaires, urinalysis, visual assessment, anthropometric measurements, dental examination, pediatric and otorhinolaryngological examination, and parental counseling. The medical questionnaire was mailed to each household in advance, and recipients were requested to complete and bring it on the day of the health checkup. The medical questionnaire and examination results of the 3‐year‐old health checkups were stored in a database by the Maternal and Child Health Program of Kobe City (Noda et al. 2021).

#### 2.1.2 Maternal and Child Health Program of Kobe City and Participants

**FIGURE 1:**
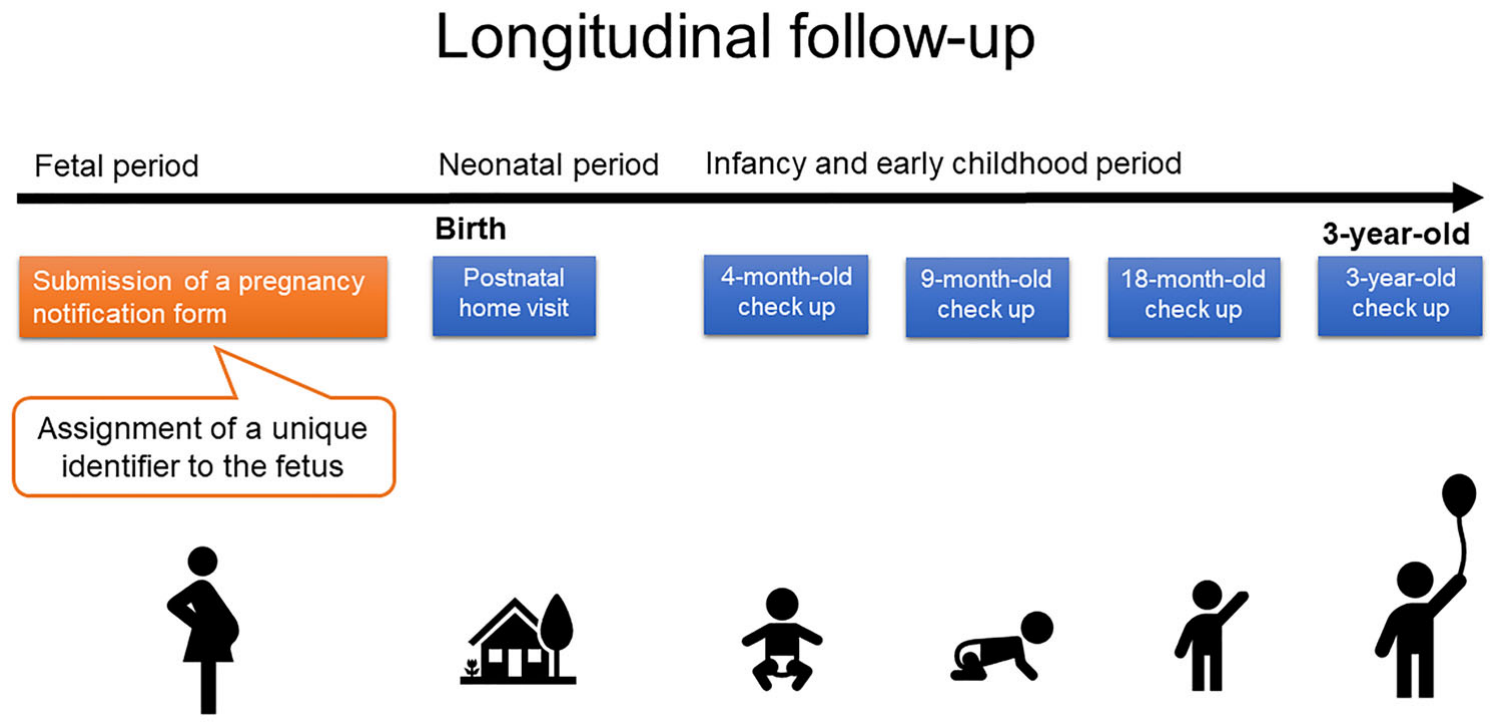
Schematic diagram of maternal and child health programs of Kobe city.

The Maternal and Child Health Program of Kobe City is a municipality‐specific initiative designed to enable longitudinal monitoring of child health and development (Figure 1). A unique identifier was assigned to each fetus when a pregnancy notification was sent to the city. This identifier allowed for longitudinal tracking through postnatal home visits and health checkups conducted at 4, 9, and 18 months, and at 3 years of age. If an individual relocated outside Kobe City, follow‐up was discontinued. Conversely, individuals who moved into Kobe City before the 3‐year‐old health checkup were assigned a unique identifier at that time. The study population comprised children who were assigned a unique identifier during the fetal period in Kobe City and were continuously followed up until the 3‐year health checkup.

#### 2.1.3 Comparison

**FIGURE 2:**
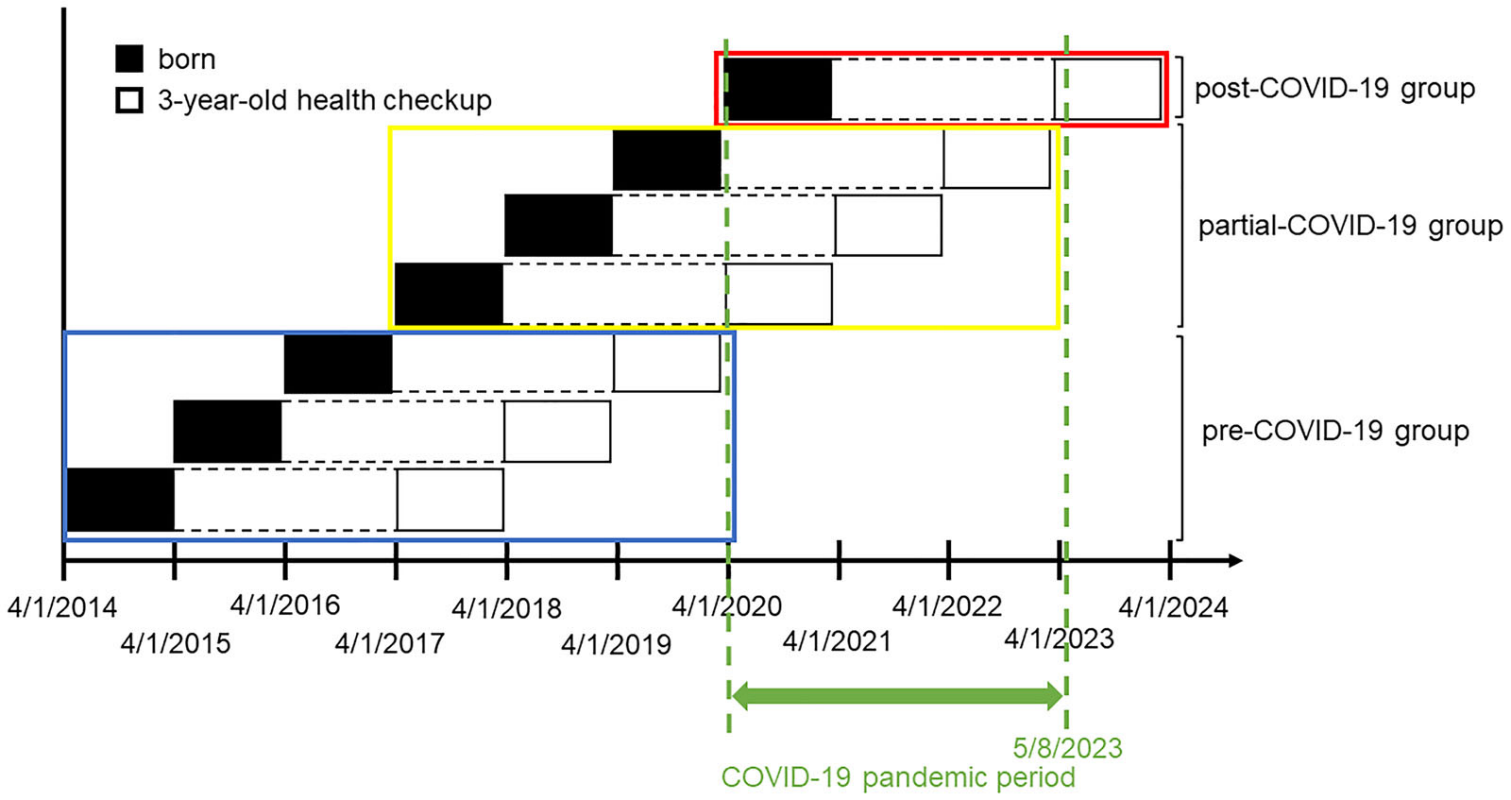
Flow chart of the participants.

To investigate the impact of the COVID‐19 pandemic on development assessed at the 3‐year‐old health checkup, we compared cohorts with varying degrees of exposure to the pandemic. Children were categorized into three groups based on their birthdates (Figure 2). The first group (pre‐COVID‐19 group) included children born between April 1, 2014, and March 31, 2017, who were entirely unaffected by the COVID‐19 pandemic until their 3‐year‐old health checkup. The second group (partial‐COVID‐19 group) comprised children born between April 1, 2017, and March 31, 2020, whose 3‐year‐old health checkup coincided with the pandemic. The third group (post‐COVID‐19 group) consisted of children born between April 1, 2020, and March 31, 2021, who were continuously exposed to the pandemic from birth until their 3‐year health checkup.

### 2.2 Measures

#### 2.2.1 Outcomes and Observational Items

We evaluated key factors associated with impairments in physical growth, motor development, and language development, including comprehension and communication at the 3‐year‐old health checkup. These factors included demographic data (such as sex, age, height, and weight) and physician‐led developmental assessments of gross motor skills, fine motor skills, and language development, including comprehension and communication. Additional data were collected from comprehensive physician evaluations and caregiver questionnaires, which assessed child‐rearing conditions and the parenting environment. All procedures were conducted in accordance with the “Guidelines for the 3‐Year‐Old Health Checkup” issued by Kobe City.

#### 2.2.2 Medical Evaluation and Conclusion by the Examining Physician

During the 3‐year‐old health checkup, the examining physician conducts a direct examination of the child. Gross motor skills are evaluated by assessing trunk muscle strength and stability, as well as coordination with lower limb muscles. Subsequently, fine motor skills are assessed to evaluate manual dexterity and control of small muscles. Finally, language development, including comprehension and communication, is assessed by asking for the name and age of the child, followed by engaging in a brief conversation (Noda et al. 2021). These assessments were conducted through clinical observations, using the “Guidelines for the 3‐Year‐Old Health Checkup” as a reference.

#### 2.2.3 Conclusion by the Examining Physician

##### 2.2.3.1 Physical Abnormalities

The examining physician checks that the height and weight of the child fall within the normal range—defined as between the 3rd and 97th percentiles based on the Japanese standard growth charts—while carefully considering overall somatic proportionality.

##### 2.2.3.2 Motor or Cognitive Abnormalities

The final diagnosis is made based on the assessment of gross and fine motor skills from the aforementioned medical evaluation (Noda et al. 2021).

##### 2.2.3.3 Concerns Regarding the Caregiving Environment

An interview is conducted by the examining physician to assess whether the caregiver has any concerns about the daily habits of the child, including wake and sleep times, eating patterns, and bowel and bladder functions.

##### 2.2.3.4 Parental Questionnaire Regarding Development

The questionnaire comprised yes‐or‐no responses and included the following items: (1) unable to jump with both feet (evaluation of gross motor skills), (2) unable to run without tripping (evaluation of gross motor skills), (3) unable to draw a circle (evaluation of fine motor function), (4) unable to understand the concept of “big” and “small” (evaluation of understanding and the ability to interpret situations), (5) limited vocabulary growth (evaluation of language development), (6) unable to produce three‐word sentences (evaluation of language development), (7) unable to engage in meaningful verbal interaction (evaluation of language development and communicative abilities), (8) unable to state the names of their playmates (evaluation of language development and social functioning), and (9) no pretend play (evaluation of play and social interaction) (Noda et al. 2021).

##### 2.2.3.5 Parental Questionnaire Responses Regarding Childcare Environments

The caregiver questionnaire also used a yes‐or‐no response format and included the following items: (1) no child‐rearing support relatives, (2) no peers or companions in parenting, (3) no playmates for their child, (4) the mother frequently plays with her child, (5) The father frequently plays with his child, and (6) profound happiness in parenting.

Explanation of each measurement instrument used in this study was shown in Table S1.

#### 2.2.4 Statistics

Descriptive data are presented as numbers (%) and means (standard deviations). Study outcomes were compared between the three groups using multivariable logistic regression adjusted for age, height, and weight at the 3‐year‐old health checkup. Overall differences between the three groups were assessed using a Wald chi‐square test in the multivariable logistic regression model. Subsequently, a pairwise comparison between the pre‐ and post‐COVID‐19 groups was conducted, which was the primary focus of this study. The Wald method was used for hypothesis testing, and odds ratios (ORs) with 95% confidence intervals were calculated for pairwise comparisons. Statistical significance was set at *p* < 0.05. All statistical analyses were performed using EZR (Jichi Medical University, Tochigi, Japan), a graphical user interface for R (The R Foundation for Statistical Computing, Vienna, Austria) (Kanda 2013).

## 3 Results

### 3.1 Number of Participants

**FIGURE 3:**
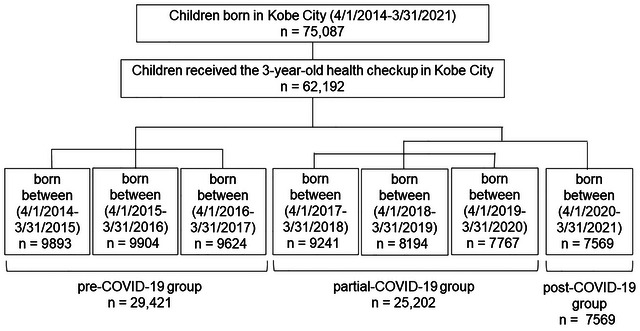
Categorization of participants based on birthdate relative to the 3‐year‐old health checkup and exposure to the COVID‐19 pandemic.

A flowchart of the participants is illustrated in Figure 3. Between April 1, 2014, and March 31, 2021, 75,087 children were born in Kobe City. Of these, 62,192 children had available birth data, such as length and weight, collected through at least one newborn home visit and completed both legally mandated health checkups at 18 months and 3 years of age (Figure 1). The pre‐, partial‐, and post‐COVID‐19 groups included 29,421, 25,202, and 7560 children, respectively (Figure 3).

### 3.2 Background of Participants

The background characteristics of the participants are presented in Table 1. Sex, gestational age, birth height, birth weight, and the rate of cesarean section did not differ between the three groups. At the time of health checkups, children in the partial‐COVID‐19 group were relatively older than those in the other groups and therefore had greater weight and height.

**TABLE 1:** Comparison of background characteristics between the pre‐, partial‐, and post‐COVID‐19 groups.

	Pre‐COVID‐19	Partial‐COVID‐19	Post‐COVID‐19
	n = 29,421	n = 25,202	n = 7,569
Sex, male	15,107 (51.3%)	12,943 (51.4%)	3,962 (51.9%)
Gestational age, weeks	38.7 (1.67)	38.7 (1.63)	38.7 (1.61)
Birth height, cm	48.6 (2.32)	48.6 (2.26)	48.6 (2.25)
Birth weight, g	3003 (425)	3009 (424)	3,014 (413)
Age at 3‐year‐old health checkup, months	40.2 (1.7)	42.0 (1.8)	39.9 (1.1)
Height at 3‐year‐old health checkup, cm	94.5 (3.6)	95.8 (3.7)	94.3 (3.5)
Weight at 3‐year‐old health checkup, kg	14.1 (1.5)	14.4 (1.6)	14.1 (1.5)

*Note*: Numbers (%) or mean (standard deviation) are shown.

### 3.3 Assessments by Physicians at the 3‐Year‐Old Health Checkup

Table 2 presents a descriptive summary of physician‐assessed motor and language development, including comprehension and communication, comparing the pre‐, partial‐, and post‐COVID‐19 groups during the 3‐year‐old health examinations. The proportion of children identified as “unable to stand on one leg” (gross motor skill) was higher in the post‐COVID‐19 group than in the pre‐COVID‐19 group (0.7% vs. 0.5%; OR 1.41, 95% CI 1.02–1.95, *p* = 0.04). In contrast, no clear difference was observed in the proportion of children identified as “unable to draw a circle” (fine motor skills). Regarding language comprehension, the proportion of children identified as having abnormalities was significantly higher in the post‐COVID‐19 group than that in the pre‐COVID‐19 group (3.6% vs. 2.1%; OR 1.72, 95% CI 1.49–1.99, *p *< 0.001). Based on the final assessment conducted by a screening physician, “physical abnormalities” were significantly less frequent in the post‐COVID‐19 group than those in the pre‐COVID‐19 group (9.9% vs. 12.1%; OR 0.79, 95% CI 0.73–0.86, *p* = 0.001). However, the proportions of children with “motor or language development abnormalities” and “concerning caregiving environments,” as judged by the examining physicians, were significantly higher in the post‐COVID‐19 group than in the pre‐COVID‐19 group (11.9% vs. 8.8%; OR 1.39, 95% CI 1.28–1.50, *p *< 0.001 for motor or cognitive abnormalities; 15.4% vs. 10.9%; OR 1.47, 95% CI 1.36–1.58, *p *< 0.001 for concerning caregiving environments).

**TABLE 2:** Comparison of neurodevelopment assessments between the pre‐, partial‐, and post‐COVID‐19 groups.

	Pre‐COVID‐19	Partial‐COVID‐19	Post‐COVID‐19	Overall	Pre‐COVID‐19 versus post‐COVID‐19	
	n = 29,421	n = 25,202	n = 7569	*p* value	Odds ratio [95% CI]	*p* value
Medical evaluation by the examining physician						
Unable to stand on one leg	134 (0.5%)	113 (0.4%)	50 (0.7%)	0.04	1.41 [1.02–1.95]	0.04
Unable to draw a circle	119 (0.4%)	114 (0.5%)	40 (0.5%)	0.32	1.27 [0.89–1.82]	0.20
Impaired language development	621 (2.1%)	769 (3.1%)	275 (3.6%)	<0.001	1.72 [1.49–1.99]	<0.001
Conclusion by the examining physician						
Physical abnormalities	3562 (12.1%)	2660 (10.6%)	747 (9.9%)	<0.001	0.79 [0.73–0.86]	0.001
Motor or language development abnormalities	2602 (8.8%)	2709 (10.7%)	900 (11.9%)	<0.001	1.39 [1.28–1.50]	<0.001
Concerns regarding the caregiving environment	3203 (10.9%)	2855 (11.3%)	1162 (15.4%)	<0.001	1.47 [1.36–1.58]	<0.001

Figure S1A–F shows a descriptive summary of the detailed birth cohort categories. This figure indicates a marked increase in the proportion of children identified with impaired language development beginning in the period affected by the COVID‐19 pandemic (Figure S1C). In contrast, the proportion of children identified as “unable to stand on one leg” (gross motor skill) showed relatively large annual fluctuations, making it challenging to attribute these changes specifically to the impact of COVID‐19 (Figure S1A). Based on the final assessment by the screening physician, trends in “physical abnormalities,” “motor or language development abnormalities,” and “concerns regarding the caregiving environment,” as concluded by the examining physician, were consistent with those summarized in Table 2 and illustrated in Figure S1D–F. However, a declining trend in physical abnormalities was observed before the COVID‐19 pandemic.

### 3.4 Responses by Caregivers to Child Assessment Questionnaires at the 3‐Year‐Old Health Checkups

Table 3 presents a descriptive summary of parental questionnaire responses regarding development, comparing the pre‐, partial‐, and post‐COVID‐19 groups at the 3‐year‐old health checkup. Regarding gross motor skills, no significant differences were observed between the three groups for “unable to jump with both feet”; however, the proportion of children classified as “unable to run without tripping” was significantly lower in the post‐COVID‐19 group than that in the pre‐COVID‐19 group (1.2% vs. 1.8%; OR 0.68, 95% CI 0.54–0.85, *p *< 0.001). In the evaluation of fine motor skills, the proportion of children unable to draw a circle was significantly higher in the post‐COVID‐19 group than that in the pre‐COVID‐19 group (3.1% vs. 2.4%; OR 1.25, 95% CI 1.08–1.46, *p* = 0.003). Cognitive development, assessed by understanding of the concepts of “big” and “small,” showed no significant differences between the three groups. For language development, the proportion of children for “limited vocabulary growth” and “unable to produce three‐word sentences” was significantly higher in the post‐COVID‐19 group than that in the pre‐COVID‐19 group (1.0% vs. 0.7%; OR 1.32, 95% CI 1.01–1.72, *p* = 0.04 for limited vocabulary growth; 4.4% vs. 3.4%; OR 1.26, 95% CI 1.11–1.43, *p *< 0.001 for unable to produce three‐word sentences). Regarding communication, the proportion of children “unable to engage in meaningful verbal interactions” was significantly higher in the post‐COVID‐19 group than that in the pre‐COVID‐19 group (3.5% vs. 2.6%; OR 1.34, 95% CI 1.16–1.55, *p* = 0.003). In addition, the proportion of children “unable to state the names of their playmates” was significantly higher in the post‐COVID‐19 group than that in the pre‐COVID‐19 group (5.5% vs. 3.9%; OR 1.40, 95% CI 1.25–1.57, *p* = 0.003). Regarding social development, which was assessed using pretend play, no significant differences were observed between the three groups.

**TABLE 3:** Comparison of parental questionnaire responses regarding development between the pre‐, partial‐, and post‐COVID‐19 groups.

	Pre‐COVID‐19	Partial‐COVID‐19	Post‐COVID‐19	Overall	Pre‐COVID‐19 versus post‐COVID‐19	
	n = 29,421	n = 25,202	n = 7569	*p* value	Odds ratio [95% CI]	*p* value
Unable to jump with both feet	311 (1.1%)	240 (1.0%)	79 (1.0%)	0.76	0.97 [0.76–1.24]	0.80
Unable to run without tripping	516 (1.8%)	383 (1.5%)	92 (1.2%)	0.002	0.68 [0.54–0.85]	<0.001
Unable to draw a circle	712 (2.4%)	617 (2.4%)	231 (3.1%)	0.01	1.25 [1.08–1.46]	0.003
Unable to understand the concept of “big” and “small”	448 (1.5%)	426 (1.7%)	128 (1.7%)	0.002	1.10 [0.90–1.34]	0.36
Limited vocabulary growth	217 (0.7%)	226 (0.9%)	75 (1.0%)	<0.001	1.32 [1.01–1.72]	0.04
Unable to produce three‐word sentences	1015 (3.4%)	923 (3.7%)	331 (4.4%)	<0.001	1.26 [1.11–1.43]	<0.001
Unable to engage in meaningful verbal interaction	763 (2.6%)	728 (2.9%)	264 (3.5%)	<0.001	1.34 [1.16–1.55]	<0.001
Unable to state the names of their playmates	1162 (3.9%)	1167 (4.6%)	417 (5.5%)	<0.001	1.40 [1.25–1.57]	<0.001
No pretend play	412 (1.4%)	409 (1.6%)	130 (1.7%)	0.005	1.22 [1.00–1.49]	0.05

Figure S2A–I shows a descriptive summary of the detailed birth cohort categories. Linguistic categories showed relatively fewer annual fluctuations and an increase in the proportion of children identified as having “limited vocabulary growth” and “unable to produce three‐word sentences” (Figure S2E–G). In addition, the proportion of children identified as “unable to state the names of their playmates” showed a similar trend (Figure S2H). In contrast, the proportion of children identified as “unable to understand the concept of ‘big’ and ‘small’” and “no pretend play” showed relatively large annual fluctuations, making it challenging to attribute the changes specifically to the impact of COVID‐19 (Figure S2D,I).

### 3.5 Responses by Caregivers to Parenting Environment Questionnaire at the 3‐Year‐Old Health Checkups

Table 4 presents a descriptive summary of parental questionnaire responses regarding the childcare environment, comparing the pre‐, partial‐, and post‐COVID‐19 groups at the 3‐year‐old health checkup. All items were significantly more frequent in the post‐COVID‐19 group than in the pre‐COVID‐19 group.

**TABLE 4:** Comparison of parental questionnaire responses regarding childcare environments between the pre‐, partial‐, and post‐COVID‐19 groups.

	Pre‐COVID‐19	Partial‐COVID‐19	Post‐COVID‐19	Overall	Pre‐COVID‐19 versus post‐COVID‐19	
	n = 29,421	n = 25,202	n = 7569	*p* value	Odds ratio [95% CI]	*p* value
No child‐rearing support relatives	3170 (10.8%)	3483 (13.8%)	1131 (14.9%)	<0.001	1.46 [1.36–1.57]	<0.001
No peers or companions in parenting	3915 (13.3%)	4450 (17.7%)	1453 (19.2%)	<0.001	1.55 [1.45–1.66]	<0.001
No playmates for their child	2585 (8.8%)	2723 (10.8%)	913 (12.1%)	<0.001	1.42 [1.31–1.53]	<0.001
The mother frequently plays with her child	15,821 (53.8%)	14,402 (57.1%)	687 (61.9%)	<0.001	1.40 [1.33–1.47]	<0.001
The father frequently plays with his child	12,810 (43.5%)	12,261 (48.7%)	3981 (52.6%)	<0.001	1.44 [1.37–1.51]	<0.001
Profound happiness in parenting	12,312 (41.9%)	11,129 (44.2%)	3515 (46.4%)	<0.001	1.20 [1.14–1.26]	<0.001

Figure S3A–F illustrates the detailed birth cohort categories. The proportion of children identified as having “no peers or companions in parenting” and “the mother and father frequently engaged in play with their child” showed a gradual upward trend, even within the pre‐COVID‐19 group (Figure S3B,D,E). However, the proportion of caregivers reporting “no playmates for their children” increased following the onset of the pandemic.

## 4 Discussion

Social interaction is essential for language learning for infants and toddlers, as language is generally acquired through interaction with others (Kuhl 2004). Adequate linguistic input is crucial, as evidenced by cases of severe neglect, where language acquisition is impaired (Sylvestre et al. 2023). In this study, our findings showed that language development was delayed in the cohort of 3‐year‐old children exposed to the COVID‐19 pandemic. Some factors may explain this delay. The proportion of children with delayed vocabulary development was 1.0% in the fully exposed cohort, compared with 0.7% in the pre‐pandemic cohort. Although this difference may appear small when viewed solely in terms of percentages, an increase from 0.7% to 1.0% corresponds to a relative increase of approximately 1.34‐fold. Given that the fully exposed cohort included approximately 7000 children, this translates to an increase from approximately 49 to 70 affected children.

Language development progresses in stages. By 4–5‐months, infants can recognize frequently heard words in their language‐learning environments (Mandel et al. 1995); they begin to vocalize by 5 months, and typically engage in babbling by approximately 7 months (Kuhl 2004). Between 6 and 9 months old, they can recognize the names of several common nouns (Bergelson and Swingley 2012). Infants generally begin to articulate their first meaningful word at approximately 12 months old (Kuhl 2004). By 18 months, word accumulation accelerates, and by 24 months, toddlers commonly start combining words into two‐word phrases (Fenson et al. 1994). Between 24 and 30 months, children acquire grammatical abilities (Fenson et al. 1994). Adequate caregiving and linguistic input are crucial, as evidenced by cases of severe neglect, where language acquisition is impaired (Sylvestre et al. 2023). Social interaction is essential for language learning for infants and toddlers, as language is generally acquired through interaction with others (Kuhl 2007). During the first 5 years of life, the brain undergoes accelerated development and growth, and early experiences play a crucial role in shaping neural architecture, function, and gene expression (Hamadani et al. 2025; Johnson 2001). Language assessment at the 3‐year‐old health checkup is relatively feasible because most children at this age can make metalinguistic judgments and produce metalinguistic responses in structured tasks, with overall metalinguistic performance improving with increasing age in months (Randall et al. 1974).

Language development in children is fostered through verbal interactions with caregivers. Previous studies have reported that the amount of verbal interaction with parents positively influences subsequent language development (Weisleder and Fernald 2013; Newman et al. 2016; Rowe 2012; Wengman and Forssman 2025; Luchkina and Xu 2024). These findings suggest that language input from caregivers, tailored to the developmental stage of the child, plays a crucial role in promoting language acquisition. However, language development in children is also fostered through play with peers in group environments such as childcare facilities. A substantial body of research has investigated the developmental links between cognition and play in peer interactions (Toseeb et al. 2020; Yogman et al. 2018; Brownell et al. 2006). Our study reports a reduction in the number of friends at 3 years of age, which may have contributed to delays in language acquisition.

In addition, teacher‐child interactions and neighborhoods have several implications for stimulating language use and development of children in daycare settings (Girolametto et al. 2000; Howard et al. 2014). Similar to our results, a study in Japan reported that the negative impact of the pandemic on language development was more pronounced in children cared for at home than in those attending nursery school (Matsuo et al. 2024). Collectively, this evidence suggests that decreased social engagement is a key factor influencing language development in early childhood, although some caregivers may have compensated by providing more attention and time to their children.

Furthermore, for children who attend daycare, factors such as social distancing and silent eating may have impeded language development. Infants efficiently perceive and process speech by visually attending to the mouth of the speaker and acquiring new vocabulary through vocal imitation. This implies that the frequent use of masks during the COVID‐19 pandemic may also have affected the language development of children because speech perception involves integrating “visual cues,” such as lip movements, and “auditory signals” during interactions.

Some studies have reported on the impact of COVID‐19 on motor development. However, the effects of COVID‐19 on gross motor skills remain unclear. For example, one study reported that Portuguese children aged 12 months exhibited significantly worse global and gross motor skills after 3 years of COVID‐19 confinement (Rebelo et al. 2024). Another study in rural Bangladesh reported that the COVID‐19 pandemic detrimentally affected cognitive and motor skills, as well as behavior of 20‐month‐old children in rural Bangladesh (Hamadani et al. 2025). However, no effect on overall motor performance was reported in 5‒6‐year‐old children in Germany (Kotzsch et al. 2025). Regarding motor skills, no impact of the COVID‐19 pandemic was observed on fine motor skills in our study. However, for gross motor skills, clinical evaluations by physicians and family questionnaires yielded opposing findings. The considerable interannual variability, particularly in gross motor skills, as illustrated in the year‐by‐year graphs, may have contributed to the challenges in identifying definitive underlying causes despite the relatively large cohort.

Our findings revealed that an increasing number of mothers lack support systems for parenting advice. In Japan, the rise of nuclear family structures and COVID‐19 social distancing measures have reduced opportunities for community‐based communication (Aggarwal et al. 2024). The COVID‐19 lockdown negatively affected maternal and child healthcare services and disrupted healthcare‐seeking behavior (Aggarwal et al. 2024). In addition, school closures have been associated with mental health problems in both children and parents (Kishida et al. 2021; Chaabane et al. 2021). Isolated mothers lacking parenting support often experience increased anxiety and burden related to child‐rearing (Arimoto and Murashima 2007; Arimoto and Tadaka 2021). Addressing maternal feelings of isolation through preventive measures—which may include promoting parenting support and fostering supportive community environments—is crucial. Although the COVID‐19 group showed a significant decrease in caregiving assistance, similar trends were observed in the pre‐COVID‐19 group, suggesting that factors beyond the pandemic contributed to this decline. Our findings indicate an increase in the amount of playtime with fathers, likely attributable to the increase in remote work during the COVID‐19 pandemic. This increased paternal involvement in childcare may have positively contributed to the parenting experiences of mothers, potentially leading to an increase in maternal satisfaction with childcare.

This study has some limitations. First, it was conducted in a single city, making it unclear whether the findings can be generalized to other cities or countries with different cultural backgrounds. Second, although health checkups were performed based on the standardized manual provided by Kobe City, variability in the clinical skills of the examining physicians may have influenced the results. Since validated assessment scales were not utilized, cutoff values could not be established. These issues may reflect inherent limitations of studies that utilize routinely collected data from public health programs; however, such studies enable the observation of large population‐based cohorts. In practice, a large number of children must be evaluated within a limited time frame. The developmental assessment method may employ relatively low thresholds to minimize the risk of missing children who require support within a limited assessment time. Even if the assessment is based on whether a child exceeds or falls below a relatively low predefined threshold, children who do not meet the threshold are realistically considered to require developmental interventions, such as language and communication support through early intervention programs. In Kobe City, the 3‐year‐old health checkup is conducted for all children at that age, which helps minimize selection bias. However, if some children migrated from rural areas, their exposure to COVID‐19 and related experiences may have differed from those of children who had continuously resided in a large metropolitan area such as Kobe City. Accordingly, children who moved to Kobe during the follow‐up period may have been affected by the COVID‐19 pandemic differently from those who did not relocate. In addition, the analysis was limited to adjustment for age, height, and weight at the 3‐year health checkup, as other potential confounding variables, such as care‐giver factors, the presence of siblings and birth order were not collected in our database.

A key strength of these data is that the same interview items are used for all children attending the health check‐ups, and physicians are provided with a standardized manual to minimize variability in clinical assessments. Furthermore, in Kobe City, the possibility of compositional changes in the sample cannot be ruled out; however, to the best of our knowledge, no major societal or healthcare‐related changes were observed during the observation period other than the COVID‐19 pandemic. Similarly, no substantial changes in maternal characteristics were observed in Kobe City during this period that could have effects comparable in magnitude to the impact of the COVID‐19 pandemic. Information regarding childcare exposure, including distinctions between home‐based care and institutional childcare, was not available in the Kobe City dataset used in this study. In addition, caregiver‐reported questionnaires may be less accurate than physician assessments, as the measures were not validated and may be subjected to recall bias. This study has several strengths, including a relatively large sample size, which enhances the robustness of the findings. In addition, the generally good nutritional status (as Japan is a high‐income country) of the participants minimizes potential confounding effects related to malnutrition. Finally, we highlight caregiver education as an important determinant of developmental outcomes.

## 5 Conclusion

The COVID‐19 pandemic was associated with changes in language development among 3‐year‐old children and the environment of their caregivers. Our findings indicate a decline in social interactions outside the family alongside a strengthening of intrafamilial orientation, which may be linked to delays in language development of children. Given the scarcity of similar studies, further studies are needed in this area.

## Author Contributions

**Kazuki Suemune**: conceptualization, methodology, software, data curation, investigation, validation, formal analysis, writing – original draft. **Hiroshi Yamaguchi**: writing – original draft, conceptualization, methodology, software, data curation, supervision, investigation, validation, formal analysis, visualization, project administration. **Masahiro Nishiyama**: conceptualization, methodology, software, data curation, investigation, validation, formal analysis, supervision, writing – review and editing, project administration. **Hiroki Mishina**: conceptualization, methodology, data curation, investigation, visualization, resources, writing – review and editing, supervision. **Aoi Kawamura**: writing – review and editing, conceptualization, investigation, supervision. **Yuki Kyono**: conceptualization, methodology, writing – review and editing, supervision, investigation, validation. **Hiroaki Nagase**: conceptualization, investigation, validation, methodology, resources, formal analysis, supervision, writing – review and editing. **Shizuka Oikawa**: supervision, conceptualization, investigation, writing – review and editing. **Kandai Nozu**: conceptualization, methodology, writing – review and editing, supervision, investigation. **Takumi Imai**: conceptualization, methodology, writing – review and editing, supervision, validation, formal analysis, data curation. **Sae Murakami**: methodology, visualization, software, data curation, writing – review and editing, supervision.

## Funding

The authors have nothing to report.

## Ethics Statement

The study was approved by the Kobe City Ethics Committee (approval number: 201915). All study procedures were conducted in accordance with the relevant institutional guidelines and regulations and the Code of Ethics of the World Medical Association (Principles of the Declaration of Helsinki).

## Consent

Informed consent was waived because of the retrospective nature of the study. However, a summary of the study was disclosed on the public departmental website, allowing individuals to opt‐out if they did not wish to participate.

## Conflicts of Interest

The authors declare no conflicts of interest.

Hiroshi Yamaguchi: hiyamgu@med.kobe-u.ac.jp

Hiroki Mishina: hiroki_mishina@city.kobe.lg.jp

## Supporting information




**Supporting Figure**: brb371434‐sup‐0001‐FigureS1.tif

**Supporting Figure**: brb371434‐sup‐0002‐FigureS2.tif

**Supporting Figure**: brb371434‐sup‐0003‐FigureS3.tif

**Supporting Table**: brb371434‐sup‐0001‐TableS1.tif

## Data Availability

The data that support the findings of this study are available from Kobe City, but restrictions apply to their access as they were used under license for the current study, and thus, are not publicly available. Data may, however, be obtained from the corresponding author, upon reasonable request, and with the permission of Kobe City. The criterion for data access permission requires that the applicant be an approved research institution in Kobe City. Once this has been met, no exclusion criteria are applied. A data use agreement must be concluded prior to access. For inquiries, please contact one of the following:
