# Supplementary figures and images for: Impact of the COVID‐19 Pandemic on Child Development and Caregiving: A 7‐Year Repeated Cross‐Sectional Study of 3‐Year‐Old Children in Kobe City, Japan
Source: Brain Behav. 2026 Apr 22;16(4):e71434. doi: 10.1002/brb3.71434 (PMC13103466; doi:10.1002/brb3.71434)

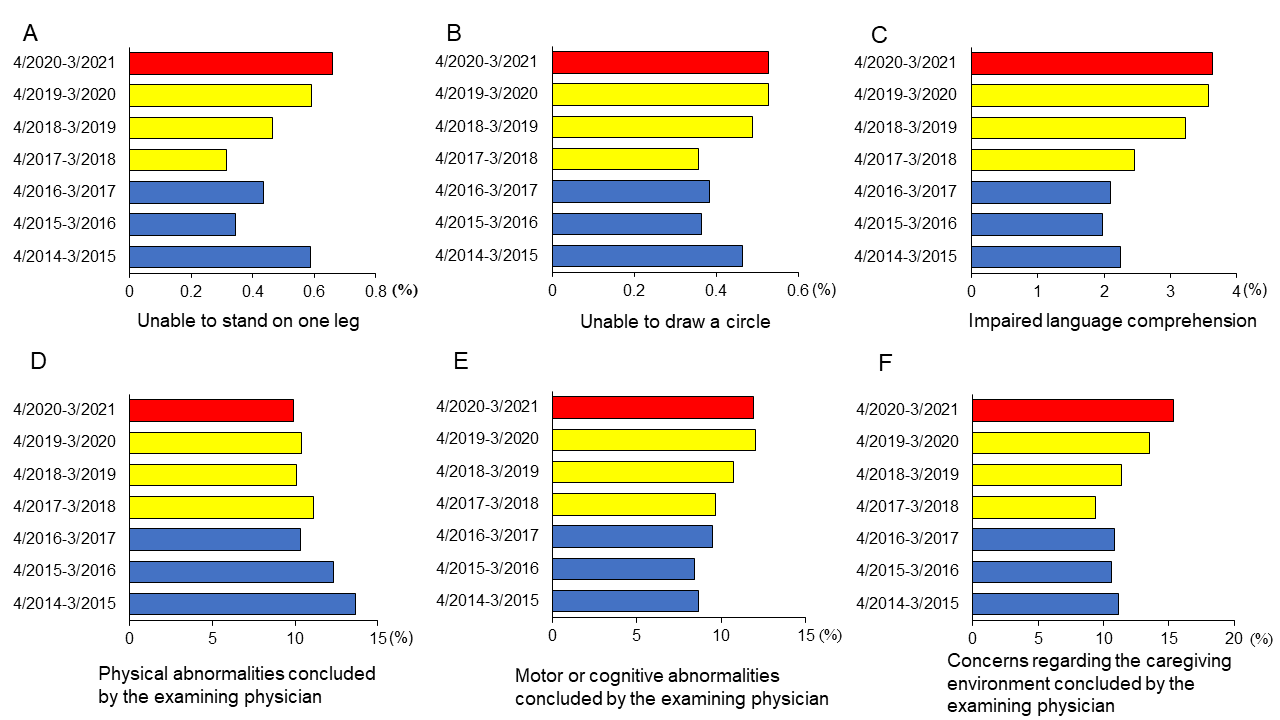

Supplement: Supplementary file 1 — Supporting Figure: brb371434‐sup‐0001‐FigureS1.tif [file BRB3-16-e71434-s002.tif]

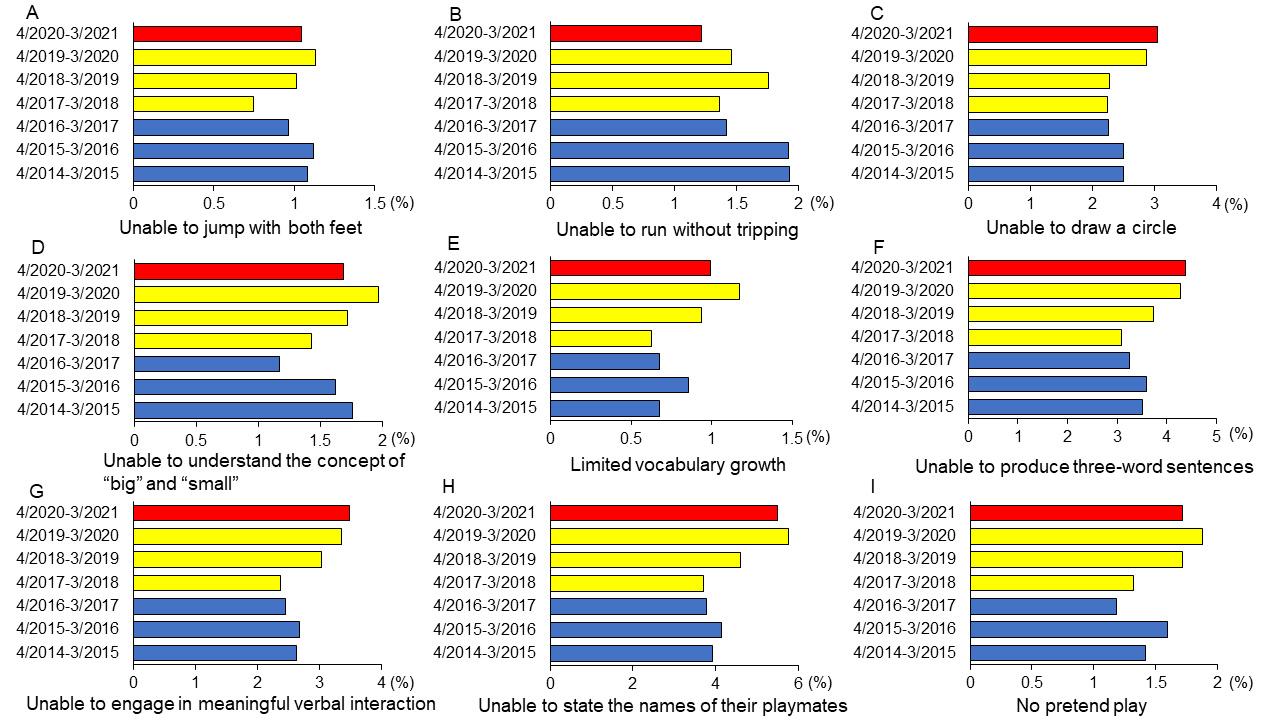

Supplement: Supplementary file 2 — Supporting Figure: brb371434‐sup‐0002‐FigureS2.tif [file BRB3-16-e71434-s003.tif]

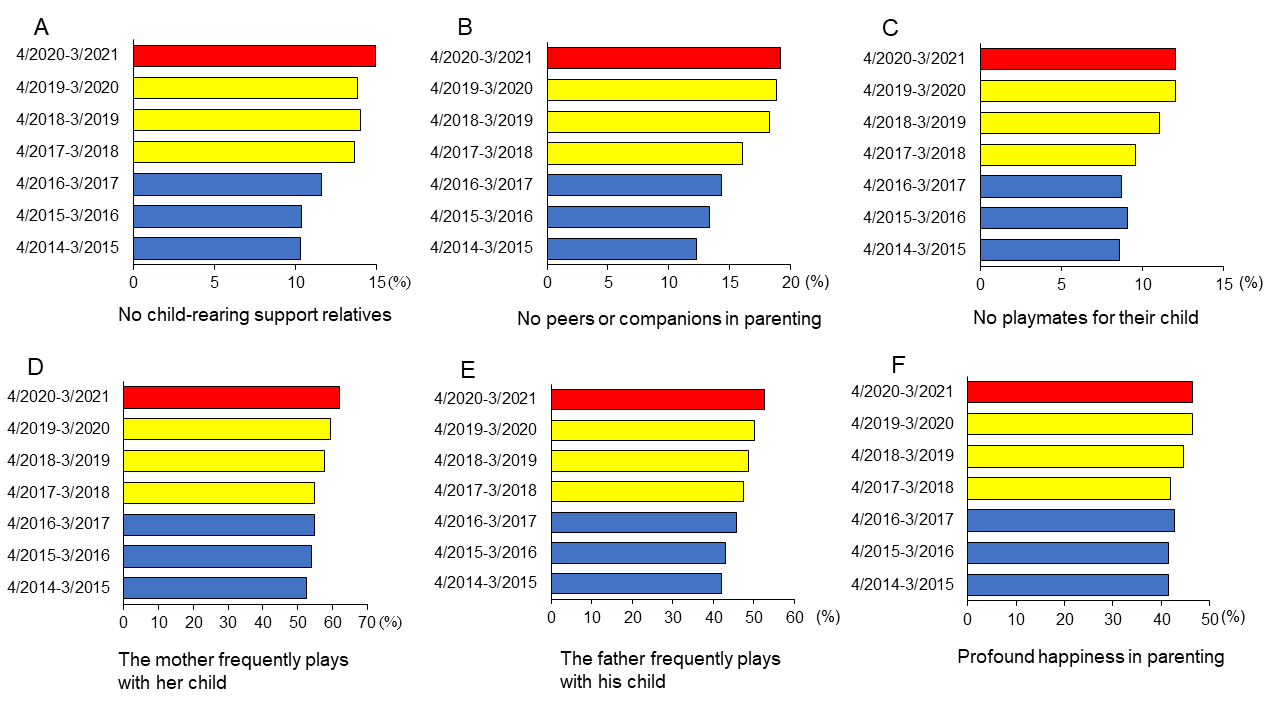

Supplement: Supplementary file 3 — Supporting Figure: brb371434‐sup‐0003‐FigureS3.tif [file BRB3-16-e71434-s004.tif]
