# Supplementary material for: Impact of the COVID‐19 Pandemic on Child Development and Caregiving: A 7‐Year Repeated Cross‐Sectional Study of 3‐Year‐Old Children in Kobe City, Japan
Source: Brain Behav. 2026 Apr 22;16(4):e71434. doi: 10.1002/brb3.71434 (PMC13103466; doi:10.1002/brb3.71434)
Supplement: Supplementary file 4 — Supporting Table: brb371434‐sup‐0001‐TableS1.tif [file BRB3-16-e71434-s001.docx]

| **Table S1. Explanation of each measurement instrument used in this study.** | |
| --- | --- |
|  |  |
| **Measurement item** | **Explanation** |
| **Medical evaluation by the examining physician** |  |
| Unable to stand on one leg | To assess gross motor skills, such as trunk muscle strength and stability, as well as coordination with lower limb muscles. |
|  |  |
| Unable to draw a circle | To assess fine motor skills, evaluating manual dexterity and the control of small muscles. |
|  |  |
| Impaired language comprehension | assessed by asking for the individual's name and age, followed by engaging in a brief conversation |
|  |  |
| **Conclusion by the examining physician** |  |
|  |  |
| Physical abnormalities | A physician's examination findings indicating that the height and weight fall within the normal range, defined as above the 3rd percentile and below the 97th percentile according to the Japanese standard growth charts, with careful consideration given to overall somatic proportionality |
|  |  |
| Motor or cognitive abnormalities | The final diagnosis is made based on the assessment of gross motor and fine motor skills from the aforementioned medical evaluation. |
|  |  |
| Concerns regarding the caregiving environment | An interview is conducted to assess whether the caregiver has any concerns about the child's daily habits, including wake and sleep times, eating patterns, and bowel and bladder functions |
| **Parental questionnaire regarding development** |  |
|  |  |
| Unable to jump with both feet | Evaluation of gross motor skills |
|  |  |
| Unable to run without tripping | Evaluation of gross motor skills |
|  |  |
| Unable to draw a circle | Evaluation of fine motor function |
|  |  |
| Unable to understand the concept of “big” and “small” | Evaluation of understanding and the ability to interpret situations |
|  |  |
| Limited vocabulary growth | Evaluation of language development |
|  |  |
| Unable to produce three-word sentences | Evaluation of language development |
|  |  |
| Unable to engage in meaningful verbal interaction | Evaluate language development and communicative abilities |
|  |  |
| Unable to state the names of their playmates | Evaluate language development and social functioning |
|  |  |
| No pretend play | Evaluate play and social interaction |
|  |  |
| **Parental questionnaire responses regarding childcare environments** |  |
|  |  |
| No child-rearing support relatives | Assess whether there are any individuals assisting with childcare |
|  |  |
| No peers or companions in parenting | Assess whether there are any individuals assisting with childcare |
|  |  |
| No playmates for their child | Assessment of whether the child has playmates |
|  |  |
| The mother frequently plays with her child | Evaluate the mother's engagement with her child |
|  |  |
| The father frequently plays with his child | Evaluate the father's engagement with her child. |
|  |  |
| Profound happiness in parenting | Evaluate the parent's emotional attitude towards childcare |
|  |  |
